# Validation of the Bangla version of the Communication Skills Attitude Scale with the medical students of Bangladesh

**DOI:** 10.1002/hsr2.2274

**Published:** 2024-08-01

**Authors:** Mohammad Aminul Islam, Maskura Benzir, Md. Kaoser Bin Siddique, Md. Abdul Awal, Mohiuddin Ahsanul Kabir Chowdhury, Taufique Joarder

**Affiliations:** ^1^ Department of Media Studies and Journalism University of Liberal Arts Bangladesh Dhaka Bangladesh; ^2^ Department of Anatomy TMSS Medical College Bogura Bangladesh; ^3^ Research, Planning & Development Department TMSS Grand Health Sector Bogura Bangladesh; ^4^ Department of Public Health Varendra University Rajshahi Bangladesh; ^5^ Associate Professor of Public Health Asian University for Women Chittagong Bangladesh; ^6^ Global Health Evaluation SingHealth Duke‐NUS Global Health Institute Singapore Singapore

**Keywords:** Bangladesh, communication in healthcare, communication skills, CSAS‐Bangla, medical student

## Abstract

**Background:**

Effective communication skill of physicians is an important component of high‐quality healthcare delivery and safe patient care. Communication is embedded in the social and cultural contexts where it takes place. An understanding of medical students' attitudes and learning communication skills would help to design and deliver culturally appropriate medical education. The Communication Skills Attitude Scale (CSAS) is a widely used and validated tool to measure the attitude of medical students toward learning communication skills in different populations, settings, and countries. However, there is no culturally adapted and validated scale in Bangla in the Bangladesh context. This study aims to culturally adapt the CSAS into Bangla, and validate it in a cohort of medical students in Bangladesh.

**Methods:**

This study used a cross‐sectional survey design to collect data from purposively selected 566 undergraduate medical students from the Rajshahi division. The survey was conducted from January to December 2023. Descriptive statistics like frequency distribution and measures of central tendency were used to measure perception regarding communication skills. The sample adequacy was measured through the Kaiser–Meyer–Olkin test. The internal consistency of the items was identified using Cronbach's alpha (*α*) coefficients.

**Result:**

The results of the study show that the Bangla version of the scale is feasible, valid, and internally consistent in the context of a developing country, Bangladesh. The overall internal consistency of the Bangla version is good since the value of Cronbach's alpha (*α*) is 0.882. For PAS, the internal consistency is 0.933. While, for NAS, the value is 0.719. The item‐wise average scores in the PAS indicate that female medical students are more willing to learn communication skills compared with male students (*α* = 0.933). While, the scores in the NAS indicate that the male students tend to have more negative attitude toward learning communication skills compared with female students (*α* = 0.719).

**Conclusion:**

The CSAS‐Bangla is a valid and reliable tool for assessing communication skill attitudes among Bangla speaking medical students. This scale can be used in future studies to measure the attitude of students, designing and evaluating communication skills training programs in medical colleges.

## 1 INTRODUCTION

Effective communication skill of physicians is an important component of high‐quality healthcare delivery and safe patient care.^1^ In a medical context, communication skills have two dimensions—social and clinical. From a social perspective, effective communication skills of physicians facilitate building a relationship of trust and respect,^2^, ^3^, ^4^, ^5^, ^6^ exchange of information and emotion about disease and illness, and empower patients in decision‐making.^7^ On the other hand, from the clinical perspective, communication skills improve health outcomes,^8^, ^9^ adherence to medical recommendations,^10^, ^11^ and patient satisfaction.^12^, ^13^, ^14^, ^15^, ^16^, ^17^, ^18^ A physician has to communicate effectively not only with patients but with colleagues, nurses, administrators, relatives of patients and news media.^1^ Because, any healthcare delivery is a team effort, effective communication with colleagues also contributes to a reduction of professional stress and burnout among healthcare professionals,^19^, ^20^, ^21^ making fewer errors in clinical decision‐making,^22^, ^23^ and health literacy of patients.^24^ So, a physician should have interpersonal communication skills,^25^, ^26^ and appropriate nonverbal communication skills—silence, facial expression, touch, and closer physical proximity.^27^, ^28^ Improved nonverbal communication skills enhance active listening and help to develop empathy and intuition among physicians.^29^ A study found that medical students' caring behavior is strongly associated with their attitudes toward communication skills.^30^, ^31^ Another study in South Korea found that medical students' patient‐centered attitude and empathic concern are correlated with their attitude toward learning communication skills.^32^ This attitude is influenced by the academic curriculum of medical schools. In a 12‐year‐long research, Gude et al.^33^ found that an emphasis on communication skills in the academic curriculum would increase positive attitudes toward learning communication skills among medical students compared with students from the traditional curriculum.

The Communication Skills Attitude Scale (CSAS) is a widely used tool to measure the attitude of medical students toward communication skills. The scale was developed by Rees et al.^34^ It has widely been used and validated in different populations, settings and countries such as Nepal,^35^ the UK^36^ India,^37^ China^38^ Malaysia,^39^ Korea,^40^ Iran,^41^ Germany,^42^ Poland,^43^ and Turkey.^44^ There are two trends in the studies on the CSAS. Like the original CSAS,^34^ some studies stick to two factorial structures,^44^, ^45^ and some studies have used a different subscale dimension.^46^, ^47^ For example, Anvik et al.^48^ applied the CSAS differently. They identified three factors—learning, importance, and respect—that influence medical students' attitudes toward learning communication skills. The learning factors refer to the student's feelings about the way communication skills are taught. The “importance” factors describe students' attitudes and values toward the importance of having communication skills for doctors. On the other hand, “respect” factors indicate students' feelings about whether good communication skills may help them respect patients and colleagues.

In Bangladesh, the medical education system consists of public, and private medical colleges. There are 109 medical colleges, with 37 public and 72 private medical colleges in the country. The total admission capacity of the institutions is around 14,000 students per year. To the best of our knowledge, no culturally adapted and validated scale is available in Bangla to evaluate medical students' attitudes toward learning communication skills. Against this backdrop, the objectives of this study are as follows:

1. To culturally adopt the CSAS into Bangla.
2. To validate CSAS in a cohort of medical students in Bangladesh.

## 2 METHODS

### 2.1 Study design and setting

This study used a cross‐sectional survey design with a convenience sample of undergraduate students at medical colleges—public and private located in the Rajshahi division, northern parts of the country. The survey was conducted online from January to December 2023. Data were collected from six medical colleges—there were public and three were private. The public medical colleges include Rajshahi Medical College, Shaheed Ziaur Rahman Medical College, Pabna Medical College, and private medical colleges include Islami Bank Medical College, Rasjshai, TMSS Medical College, Bogura, Barind Medical College, Rajshahi. Although this study selected only one division, students from all over the country enroll at the medical colleges for studying medicine.

### 2.2 Participants and sampling method

Based on the objective of the study, this study used purposive sampling method to collect data from different public and private medical colleges approved by the Bangladesh Medical & Dental Council (BMDC), the state body that is responsible for establishing and maintaining quality of medical education and recognition of medical qualifications in Bangladesh. The students enrolled only undergraduate level students who were studying at Bachelor of Medicine and Bachelor of Surgery (MBBS). The study considered the following recognized sample size determination formula:

n=z21−α/2(pq)/d2,

Here, *p* = 0.5 (50%), *q* = 0.5 (50%), z = 1.96 (95% CI), *d* = 0.05 (5%), *n* = 384.

where, *p* is the prevalence of outcome variables, *q* = 1‐*p*, Z value = 1.96 for 95% confidence interval, *α* the level of significance = 5%, *d* the desired margin of error or precision = 0.05 (5%), *n* the required minimum sample size, Required minimum sample size *n* = 384.

However, this study reached 566 participants. The total number of students at the selected medical colleges were 4625. We aimed at reaching at least 10% of the population. However, we managed to reach 12.23% of the population for the study.

### 2.3 Instrument

In this study, we used CSAS. It is a 26‐item 2‐factor scale with 13 items on each subscale. The factor I under Positive Attitude Scale (PAS) represented positive attitudes toward communication skills learning, and factor II under Negative Attitude Scale (NAS) represented negative attitudes. The participants were asked to rate their opinion on the statements such as “To be a good doctor, I must have good communication skills” on a scale where 1 indicated strongly disagree, 2 indicated disagree, 3 indicated neutral, 4 indicated agree, and 5 indicated strongly agree.

## 3 ADAPTATION OF CSAS

The original CSAS was in English (see Annex‐I), and it was adapted into Bangla by following standard forward and backward translation. The forward translation from English to Bangla was performed by an expert translator. The expert had a master's degree in English language and literature and had more the 15 years of experience in professional translation. He was not aware of the research. Another forward translation was performed by a medical student. He was also fluent in both English and Bangla. Later, both the forward translated versions were compared and contrasted by the paper's two authors—one was an expert in communication in healthcare, and had bachelor's and master's degrees in communication, and another was a trained physician. After addressing and accommodating differences, inconsistencies, and variances, they compiled and merged both translations into a single Bangla‐translated version. Then another medical graduate and public health expert back‐translated the tool into English. All the translators are native speakers of Bangla and fluent in English. The translators were instructed to use simple language and to capture the meaning of the item rather than perform a literal word‐by‐word translation. The back‐translation version was compiled by following similar procedures to the forward translation. Later, all four versions were evaluated by an expert committee formed for this study. The expert committee reviewed and suggested the final adaptation of the instrument (see Annex II). The reviewed version was used for the pretest among 52 respondents of the study.

### 3.1 Data collection and quality assurance

First, survey questionnaire (see Annex III) was distributed among some faculty members of the selected medical colleges. The faculty members requested their students to fill up the questionnaire in different classes of the selected medical colleges. The data were stored electronically with utmost secrecy.

### 3.2 Measurements and statistical methods

The data were analyzed using the statistical software Stata version 17.0. The characteristics of the study participants and their perception regarding communication skills were analyzed through descriptive statistics like frequency distribution and the measures of central tendency. The attitudinal scores for both PAS and NAS were presented with mean ± Standard Deviation. The sample adequacy was measured through the Kaiser–Meyer–Olkin test. The test statistic was marked as highly adequate if the value is more than 0.8, moderately adequate if the value is between 0.5 and 0.8, and inadequate otherwise. The internal consistency of the items was identified using Cronbach's alpha (*α*) coefficients.

### 3.3 Reliability

We measured the reliability of the Bangla version of CSAS through internal consistency, using Cronbach's *α* coefficient.

### 3.4 Ethical consideration

We obtained informed consent from the participants by explaining the study's aims, objectives, data collection and storage process, privacy, benefits, risks and rights to withdraw at any stage of the research. Ethical clarence for the study was obtained from the Public Health Foundation, Bangladesh (PHFBD‐ERC‐IP06/2023).

## 4 RESULTS

### 4.1 Characteristics of the sample

The average age of the participants was 22 (SD = 2.6) years. More than half of the total participants were female 329 (58.1%). Around half of the total participants were from private medical colleges 299 (52.8%), and the rest were from public medical colleges. The majority of 281 (49.7%) of the participants were studying between first and second year at their medical colleges. Details are shown in Table 1.

**Table 1:** Basic characteristics of the participants (*N* = 566).

Characteristics (Frequency (%)/Mean ± SD)	Male (*n* = 237)	Female (*n* = 329)	Total (*N* = 566)
Age	21.5 ± 2.4	22.6 ± 2.8	22.0 ± 2.6
Type of medical school			
Public	128 (54.0)	139 (42.3)	329 (58.1)
Private	109 (46.0)	190 (57.7)	237 (41.9)
Place of residence			
Urban	109 (46.0)	189 (57.5)	267 (47.2)
Rural	128 (54.0)	140 (42.5)	299 (52.8)
Medical education, years			
<1 year	2 (0.8)	1 (0.3)	3 (0.5)
1–2 years	91 (38.4)	190 (57.8)	281 (49.7)
2–3 years	27 (11.4)	28 (8.5)	55 (9.7)
3–4 years	64 (27.0)	60 (18.2)	124 (21.9)
4–5 years	44 (18.6)	42 (12.8)	86 (15.2)
>5 years	9 (3.8)	8 (2.4)	17 (3.0)

## 5 ATTITUDINAL SCORES IN ALL ITEMS OF THE CSAS QUESTIONNAIRE

Data in Table 2 presents information about attitudinal scores for both male and female participants in all items of the CSAS questionnaire. In the PAS, the attitude of the participants indicates that most of the medical students strongly believe that they need to have good communication skills to be a good doctor (Mean 4.50, and SD 1.04), and communication skills teaching should be more like a science subject (Mean 3.85, SD 1.19). From the table, it is evident that overall female medical students are more willing to learn communication skills compared male students as shown in the item‐wise average scores of the PAS scale. On the other hand, the NAS indicate that the students are reluctant to learn communication as they think that they do not need good communication skills to be a doctor (Mean 1.61, SD 1.11). Meanwhile, the item‐wise average scores in the NAS scale indicate that male students tend to have more negative attitude toward learning communication skills compared with female students. Details are shown in Table 2.

**Table 2:** Attitudinal scores in all items of the CSAS questionnaire (range 1–5).^34^

Items	Female	Male	Overall	*p* Value
*PAS*				
1. To be a good doctor I must have good communication skills	4.57 ± 0.97	4.41 ± 1.13	4.50 ± 1.04	0.066
4. Developing my communication skills is just as important as developing my knowledge of medicine	4.38 ± 1.05	4.18 ± 1.19	4.30 ± 1.12	0.034
5. Learning communication skills has helped or will help me respect patients	4.42 ± 1.10	4.32 ± 1.16	4.38 ± 1.13	0.288
7. Learning communication skills is interesting	4.28 ± 1.06	4.19 ± 1.07	4.24 ± 1.07	0.323
9. Learning communication skills has helped or will help facilitate my team‐working skills	4.39 ± 0.98	4.23 ± 1.16	4.32 ± 1.06	0.074
10. Learning communication skills has improved my ability to communicate with patients	4.17 ± 1.04	4.14 ± 1.07	4.16 ± 1.05	0.739
12. Learning communication skills is fun	4.14 ± 1.11	4.0 ± 1.26	4.08 ± 1.17	0.162
14. Learning communication skills has helped or will help me respect my colleagues	4.30 ± 1.02	4.08 ± 1.10	4.21 ± 1.06	0.014
16. Learning communication skills has helped or will help me recognize patients' rights regarding confidentiality and informed consent	4.22 ± 1.08	4.04 ± 1.07	4.15 ± 1.16	0.059
17. Communication skills teaching would have a better image if it sounded more like a science subject	3.86 ± 1.17	3.83 ± 1.21	3.85 ± 1.19	0.775
21. I think it's really useful to learn communication skills on a medical degree	4.31 ± 1.04	4.03 ± 1.28	4.19 ± 1.15	0.004
23. Learning communication skills applies to learning medicine	4.09 ± 1.10	3.93 ± 1.22	4.02 ± 1.15	0.119
25. Learning communication skills is important because my ability to communicate is a lifelong skill	4.40 ± 1.05	4.20 ± 1.24	4.32 ± 1.14	0.037
*NAS*				
2. I can't see the point in learning communication skills	1.69 ± 1.34	1.75 ± 1.42	1.72 ± 1.37	0.657
3. Nobody is going to fail their medical degree for having poor communication skills	2.74 ± 1.33	2.77 ± 1.35	2.76 ± 1.34	0.831
6. I haven't got time to learn communication skills	3.17 ± 1.29	3.21 ± 1.26	3.19 ± 1.28	0.751
8. I can't be bothered to turn up to sessions on communication skills	1.85 ± 1.15	1.84 ± 1.21	1.85 ± 1.18	0.943
11. Communication skills teaching states the obvious and then complicates it	2.77 ± 1.23	2.68 ± 1.29	2.73 ± 1.26	0.373
13. Learning communication skills is too easy	3.16 ± 1.17	3.22 ± 1.29	3.18 ± 1.22	0.536
15. I find it difficult to trust information about communication skills given to me by nonclinical lecturers	2.93 ± 1.25	2.80 ± 1.19	3.18 ± 1.23	0.215
18. When applying for medicine, I thought it was a really good idea ….	3.68 ± 1.24	3.38 ± 1.35	3.55 ± 1.29	0.005
19. I don't need good communication skills to be a doctor	1.61 ± 1.13	1.60 ± 1.08	1.61 ± 1.11	0.911
20. I find it hard to admit to having some problems with my communication skills	2.44 ± 1.31	2.59 ± 1.25	2.51 ± 1.29	0.180
22. My ability to pass exams will get me through medical school rather than my ability to communicate	2.84 ± 1.33	2.99 ± 1.30	2.90 ± 1.32	0.195
24. I find it difficult to take communication skills learning seriously	2.49 ± 1.28	2.44 ± 1.19	2.47 ± 1.25	0.642
26. Communication skills learning should be left to psychology students, not medical students	1.71 ± 1.21	1.79 ± 1.14	1.74 ± 1.13	0.384

## 6 SAMPLE ADEQUACY AND INTERNAL CONSISTENCY

Overall, the KMO value is 0.940 which corresponds to excellent sample adequacy. The internal consistency of the items is good since the overall value of *α* is 0.882. For PAS, the internal consistency is 0.933. While, for NAS, the value is 0.719. Details are shown in Table 3.

**Table 3:** Measures of sample adequacy and internal consistency.

Item groups	Criteria	Value
PAS	Number of items	13
	Average interitem covariance	0.680
	Cronbach *α*	0.933
NAS	Number of items	13
	Average interitem covariance	0.247
	Cronbach *α*	0.719
Overall	Number of items	26
	Average interitem covariance	0.313
	Cronbach *α*	0.882

## 7 CORRELATION BETWEEN THE FACTORS

The items are not correlated with each other within or without the groups (*p* value: <0.001). Details are shown in Table 4.

**Table 4:** Findings from Bartlett's test for sphericity to check the correlation between the factors.

Item groups	Criteria	Value
PAS	Determinant of the correlation matrix	0.000
	Chi‐square value	5422.34
	Degree of freedom	78
	*p* value	**<0.001** ^*^
NAS	Determinant of the correlation matrix	0.075
	Chi‐square value	1446.58
	Degree of freedom	78
	*p* value	**<0.001** ^*^
Overall	Determinant of the correlation matrix	0.000

*Note*: * indicate statistically significant.

**Figure 1:**
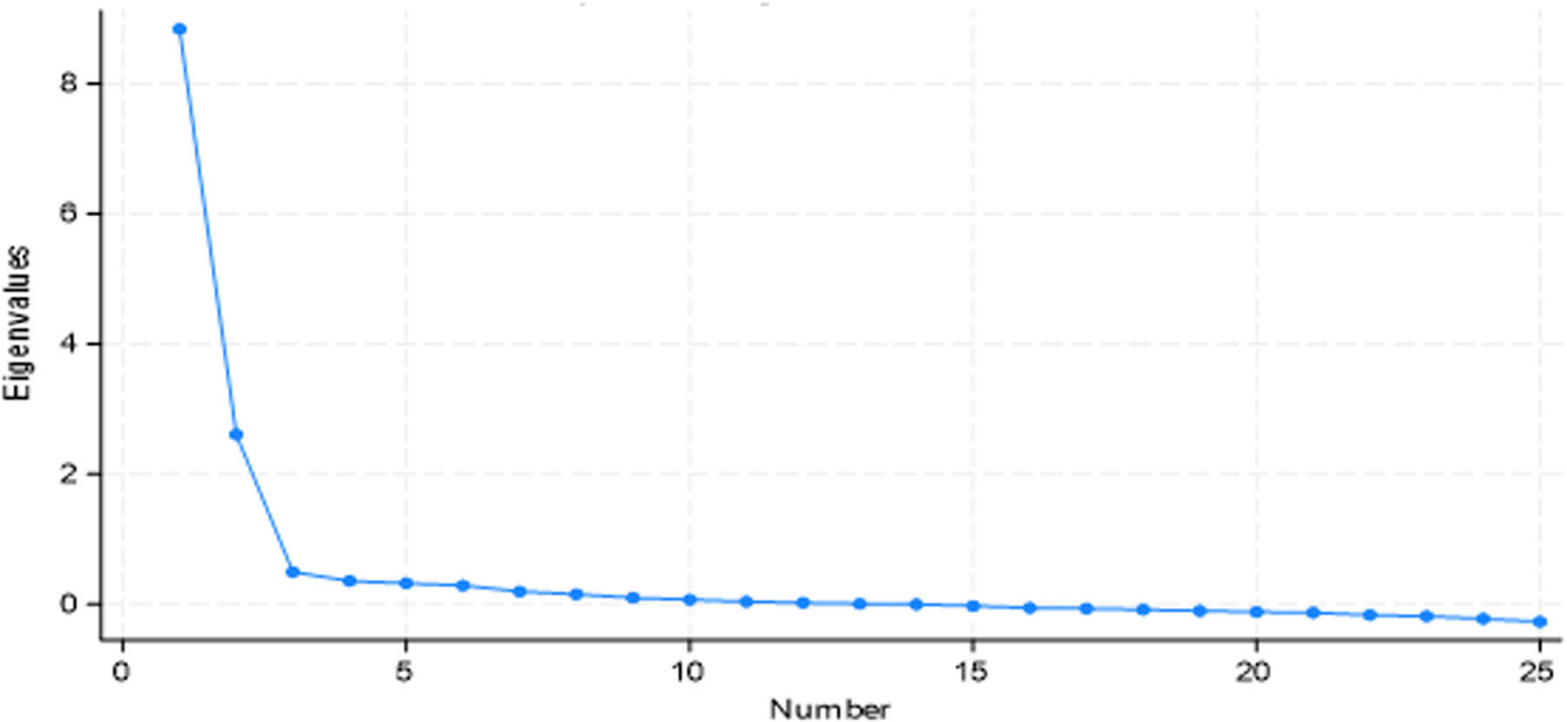
Scree plot of Eigenvalues.

Figure 1 displays a steep curve in the scree plot of the Eigenvalues, indicating that the top two or three components adequately describe the data's essence. Details are shown in Figure 1.

Table 5 shows the Eigenvalues for different factors utilized in Principal Component Analysis. Details are shown in Table 5.

**Table 5:** Eigenvalues for the factors.

Factor	Eigenvalue	Difference	Proportion	Cumulative
Factor1	8.83628	6.22634	0.7293	0.7293
Factor2	2.60994	2.11273	0.2154	0.9447
Factor3	0.49721	0.13714	0.0410	0.9858
Factor4	0.36007	0.03505	0.0297	1.0155
Factor5	0.32502	0.03456	0.0268	1.0423
Factor6	0.29046	0.09286	0.0240	1.0663
Factor7	0.19760	0.04171	0.0163	1.0826
Factor8	0.15589	0.05410	0.0129	1.0954
Factor9	0.10179	0.02844	0.0084	1.1038
Factor10	0.07335	0.03282	0.0061	1.1099
Factor11	0.04053	0.01475	0.0033	1.1132
Factor12	0.02578	0.01729	0.0021	1.1154
Factor13	0.00849	0.00943	0.0007	1.1161
Factor14	−0.00094	0.02481	−0.0001	1.1160
Factor15	−0.02575	0.02806	−0.0021	1.1139
Factor16	−0.05380	0.01012	−0.0044	1.1094
Factor17	−0.06393	0.01794	−0.0053	1.1042
Factor18	−0.08186	0.01746	−0.0068	1.0974
Factor19	−0.09932	0.01835	−0.0082	1.0892
Factor20	−0.11767	0.01075	−0.0097	1.0795
Factor21	−0.12842	0.03667	−0.0106	1.0689
Factor22	−0.16509	0.01657	−0.0136	1.0553
Factor23	−0.18166	0.03985	−0.0150	1.0403
Factor24	−0.22151	0.04488	−0.0183	1.0220
Factor25	−0.26639	–	−0.0220	1.0000

**Figure 2:**
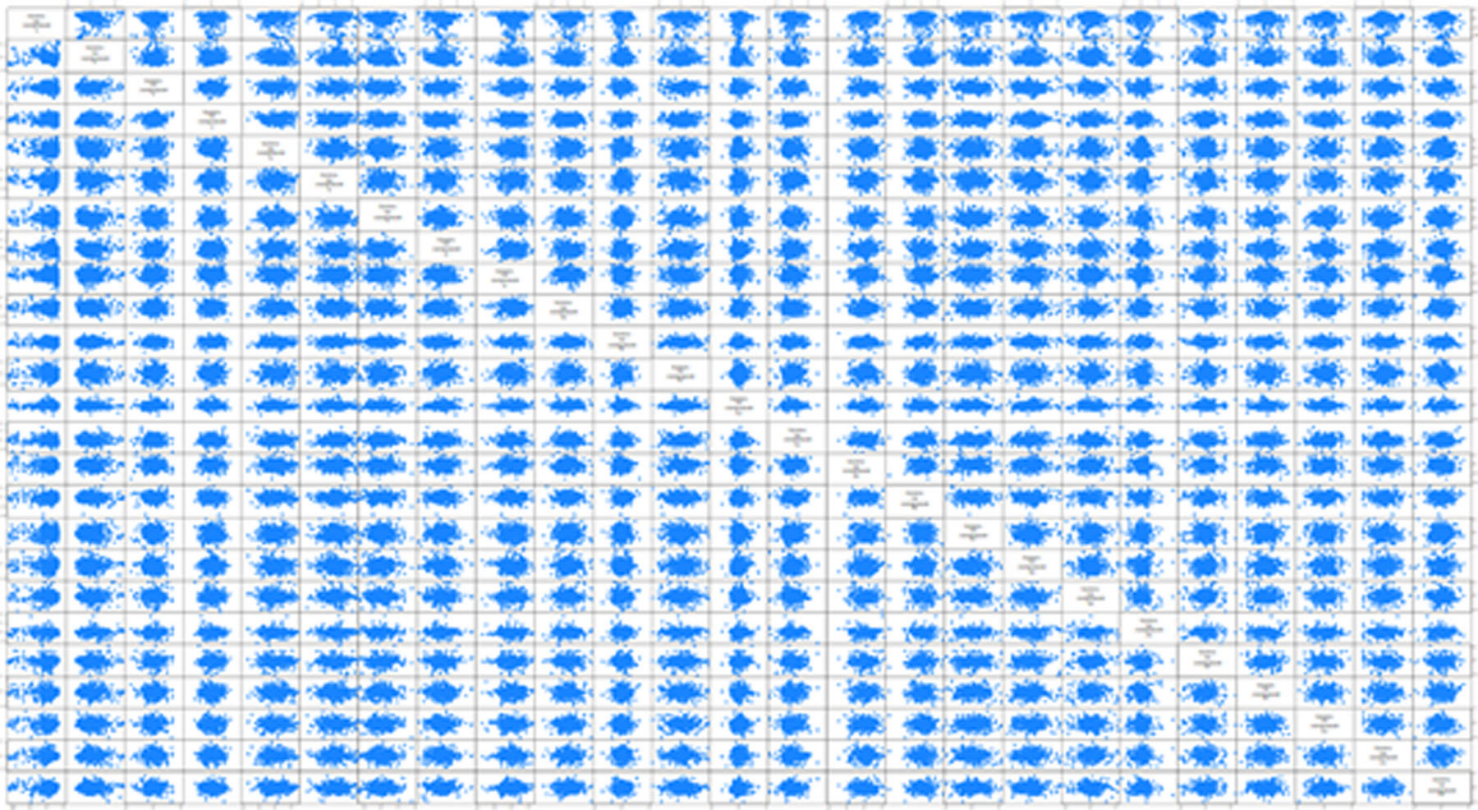
Scatter plots showing the relationship between different factors of Principal Component Analysis.

Figure 2 shows the relationship between the factors used in PCA through scatter plots. As per Figure 2 and Table 5, Factor 1 is most strongly correlated with each other component. Details are shown in Figure 2.

## 8 DISCUSSIONS

This study translated and adapted the CSAS into the Bangla language and validated it in a cohort of medical students in Bangladesh. It found that the Bangla version of the scale is feasible, valid, and internally consistent.

The overall internal consistency of the CSAS‐Bangla is 0.882, and the values of Cronbach *α* of the two subscales are 0.933 and 0.719. In terms of internal consistency, it has a higher degree of similarity with some previous studies. For example, the overall internal consistency of the Polish version was *α* 0.853,^49^ and the Iranian version 0.84.^41^

Similar to the original CSAS and some other studies that had two factorial structures, the Bangla version of the CSAS also has two components. However, several other studies in different contexts yielded more than to two subscales. For example, the Norwegian version had three subscales—“learning,” “importance,” and “respect”^48^; and the Hungarian version had seven—respect and interpersonal skills, learning, the importance of communication in the medical profession, excuse, counter, exam and overconfidence, and Persian version of the CSAS had four components—important in the medical context, excuse, learning and overconfidence.^41^ The difference in the factorial structures may be due to socioeconomic, geographic, cultural, and linguistic differences of the participants.

To the best of our knowledge, this is the first endeavor to validate the psychometric properties of CSAS in the Bangla language, and from the context of a developing country like Bangladesh. While most of the previous validation of the scale included students from a single medical college, we collected data from various medical colleges.

The results of our study have both theoretical and practical implications. Theoretically, it will contribute to the understanding of the attitude of medical students toward learning communication skills in the context of a developing country. Moreover, it will help to improve the curriculum and delivery of medical education. Thus, it will contribute to preparing future physicians equipping them with the art and craft of communication and ensuring better healthcare delivery.

However, acknowledge that this study has some limitations. First, we collected data from only a region of the country as the medical colleges we selected for data collection were located only in the northern region of Bangladesh. Second, we collected data mainly from undergraduate level medical students. Third, we did not put emphasis on collecting data from students of dental colleges. So, future studies be conducted in a large scale with a focus on medical and dental students from different levels and different medical colleges across the country. Future studies also should focus on postgraduate level students.

## 9 CONCLUSION

We conclude that CSAS‐Bangla is a valid and reliable tool for assessing communication skill attitudes among Bangla speaking medical students in Bangladesh. The psychometric properties of this tool are a novel approach to understanding the communication dynamics of preparing future healthcare professionals in the context of a developing country, Bangladesh. This scale can be used in future studies in measuring the attitude toward learning communication skills among medical Bangladesh, designing and evaluating communication skills training programs in medical colleges in the country.

## AUTHOR CONTRIBUTIONS

Mohammad Aminul Islam contributed to conceptualizing the study, designing the study, preparing data collection tools, data collection, data analysis, writing manuscript and finalizing the manuscript, and coordinating the project. Maskura Benzir contributed to designing the study and data collection. Md. Kaoser Bin Siddique contributed to writing the draft manuscript and data collection. Md. Abdul Awal contributed to conceptualizing the study and data collection. Mohiuddin Ahsanul Kabir Chowdhury contributed to designing the study, data analysis, and writing the manuscript. Taufique Joarder contributed to conceptualizing the study, writing and finalizing the manuscript, and supervising the project.

## CONFLICT OF INTEREST STATEMENT

The authors declare no conflict of interest.

## TRANSPARENCY STATEMENT

The lead author Mohammad Aminul Islam affirms that this manuscript is an honest, accurate, and transparent account of the study being reported; that no important aspects of the study have been omitted; and that any discrepancies from the study as planned (and, if relevant, registered) have been explained.

## Data Availability

The data that support the findings of this study are available to the first author of the study. However, the data is not publicly available due to privacy concerns and confidentiality agreements with participants of the study.

## 1

**Communication Skills Attitude Scale (CSAS)** ^34^ **[Original English version]**

Please read the following statements about communication skills learning. Indicate whether you disagree or agree with all of the statements according to the following scale: 1 = *strongly disagree*, 2 = *disagree*, 3 = *neutral*, 4 = *agree*, and 5 = *strongly agree*.

**Table:**

	Strongly disagree	Disagree	Neutral	Agree	Strongly Agree
	1	2	3	4	5
1. To be a good doctor I must have good communication skills	1	2	3	4	5
2. I can't see the point in learning communication skills	1	2	3	4	5
3. Nobody is going to fail their medical degree for having poor communication skills	1	2	3	4	5
4. Developing my communication skills is just as important as developing my knowledge of medicine	1	2	3	4	5
5. Learning communication skills has helped or will help me respect patients	1	2	3	4	5
6. I haven't got time to learn communication skills	1	2	3	4	5
7. Learning communication skills is interesting	1	2	3	4	5
8. I can't be bothered to turn up to sessions on communication skills	1	2	3	4	5
9. Learning communication skills has helped or will help facilitate my team‐working skills	1	2	3	4	5
10. Learning communication skills has improved my ability to communicate with patients	1	2	3	4	5
11. Communication skills teaching states the obvious and then complicates it	1	2	3	4	5
12. Learning communication skills is fun	1	2	3	4	5
13. Learning communication skills is too easy	1	2	3	4	5
14. Learning communication skills has helped or will help me respect my colleagues	1	2	3	4	5
15. I find it difficult to trust information about communication skills given to me by nonclinical lecturers	1	2	3	4	5
16. Learning communication skills has helped or will help me recognize patients' rights regarding confidentiality and informed consent	1	2	3	4	5
17. Communication skills teaching would have a better image if it sounded more like a science subject	1	2	3	4	5
18. When applying for medicine, I thought it was a really good idea to learn communication skills	1	2	3	4	5
19. I don't need good communication skills to be a doctor	1	2	3	4	5
20. I find it hard to admit to having some problems with my communication skills	1	2	3	4	5
21. I think it's really useful learning communication skills on the medical degree	1	2	3	4	5
22. My ability to pass exams will get me through medical school rather than my ability to communicate	1	2	3	4	5
23. Learning communication skills is applicable to learning medicine	1	2	3	4	5
24. I find it difficult to take communication skills learning seriously	1	2	3	4	5
25. Learning communication skills is important because my ability to communicate is a lifelong skill	1	2	3	4	5
26. Communication skills learning should be left to psychology students, not medical students	1	2	3	4	5

Scoring key: To obtain score of PAS, add scores of items 4,5,7,9,10,12,14,16,18,21,23,25 and reversed score of 22. Score of NAS will obtained by adding the scales of items 2,3,6,8,11,13,15,17,19,20,24,26 and reverse score of item number 1. Both scales range from 13 to 65, and higher scores in both represent stronger positive or negative attitudes.

## ANNEX II

**Bangla translation of the Communication Skills Attitude Scale (CSAS) [যোগাযোগ দক্ষতার প্রতি মনোভাব স্কেল‐এর বাংলা অনুবাদ]**

Please read the following statements about communication skills learning. Indicate whether you disagree or agree with all of the statements according to the following scale: 1 = *strongly disagree*, 2 = *disagree*, 3 = *neutral*, 4 = *agree*, and 5 = *strongly agree*. [রোগীর সঙ্গে যোগাযোগ [‘কথা বলা, শোনা, অঙ্গভঙ্গি, মুখোভঙ্গি ও কণ্ঠস্বর ইত্যাদি] দক্ষতা শিক্ষণ বিষয়ে নিম্নলিখিত বিবৃতিগুলো দয়া করে পড়ুন। নিচের স্কেলে উল্লেখিত যেসব বিবৃতির সঙ্গে আপনি দ্বিমত বা একমত পোষণ করেন তা নির্দেশ করুন। আর সেক্ষেত্রে টিক চিহ্ন ব্যবহার করুন। এখানে, ১= দৃঢ়ভাবে দ্বিমত পোষণ করি, ২ = দ্বিমত পোষণ করি, ৩ = নিরপেক্ষ, ৪ = একমত পোষণ করি, এবং ৫ = দৃঢ়ভাবে একমত পোষণ করি]

**Table:**

	Strongly disagree [দৃঢ়ভাবে দ্বিমত পোষণ করি]	Disagree [দ্বিমত পোষণ করি]	Neutral [নিরপেক্ষ/ নিশ্চিত নিই/বুঝতে পারছি না]	Agree [একমত পোষণ করি]	Strongly Agree [দৃঢ়ভাবে একমত পোষণ করি]
	১	২	৩	৪	৫
1. To be a good doctor I must have good communication skills [একজন ভালো ডাক্তার হতে হলে আমার ভালো যোগাযোগ দক্ষতা দক্ষতা থাকতে হবে।]	১	২	৩	৪	৫
2. I can't see the point in learning communication skills [একজন ডাক্তারের তার রোগীর সঙ্গে ভালো যোগাযোগ দক্ষতা শেখার দরকার আছে বলে আমি মনে করি না।	১	২	৩	৪	৫
3. Nobody is going to fail their medical degree for having poor communication skills [রোগীর সঙ্গে ভালো যোগাযোগ দক্ষতা না থাকার কারনে মেডিকেল কলেজের পরিক্ষায় কোনো শিক্ষার্থী অকৃতকার্য হয় না।]	১	২	৩	৪	৫
4. Developing my communication skills is just as important as developing my knowledge of medicine. [রোগীর সঙ্গে যোগাযোগ দক্ষতা বাড়ানো চিকিৎসাবিদ্যায় প্রয়োজনীয় জ্ঞান অর্জনের মতই সমানভাবে গুরুত্বপূর্ণ।]	১	২	৩	৪	৫
5. Learning communication skills has helped or will help me respect patients [রোগীর সঙ্গে ভালো যোগাযোগ দক্ষতা শেখা আমাকে রোগীর প্রতি সম্মান প্রদর্শনে সহায়তা করেছে বা ভবিষ্যতে করবে।]	১	২	৩	৪	৫
6. I haven't got time to learn communication skills [আমি রোগীর সঙ্গে ভালোভাবে যোগাযোগ দক্ষতা শেখার সময় পায়নি।]	১	২	৩	৪	৫
7. Learning communication skills is interesting [রোগীর সঙ্গে ভালোভাবে যোগাযোগ দক্ষতা শেখার বিষয়টি বেশ আকর্ষণীয়]	১	২	৩	৪	৫
8. I can't be bothered to turn up to sessions on communication skills [রোগীর সঙ্গে ভালো যোগাযোগ দক্ষতা শেখার কোনো ক্লাশ বা প্রশিক্ষণ কর্মশালয় অংশগ্রহণে আমার কোনো আগ্রহ নেই।]	১	২	৩	৪	৫
9. Learning communication skills has helped or will help facilitate my team‐working skills [রোগীর সঙ্গে ভালো যোগাযোগ দক্ষতা শেখা আমাকে অন্যের সঙ্গে একযোগে কাজ করতে সহায়তা করেছে বা করবে।]	১	২	৩	৪	৫
10. Learning communication skills has improved my ability to communicate with patients [যোগাযোগ দক্ষতা শেখার ফলে আমার রোগীর সঙ্গে যোগাযোগ সক্ষমতা বেড়েছে।	১	২	৩	৪	৫
11. Communication skills teaching states the obvious and then complicates it [যোগাযোগ দক্ষতা শিক্ষণ প্রক্রিয়ায় মূলত জানা বিষয়গুলোই তুলে ধরা হয়। কিন্তু এসব বিষয় শেখার ফলে, রোগীর সঙ্গে বলা ও শোনার স্বাভাবিক বিষয়টি বেশ জটিলও হয়ে যায়।]	১	২	৩	৪	৫
12. Learning communication skills is fun [যোগাযোগ দক্ষতা শেখার বিষয়টি বেশ আনন্দদায়ক]	১	২	৩	৪	৫
13. Learning communication skills is too easy রোগীর সঙ্গে যোগাযোগ দক্ষতা শেখার বিষয়টি বেশ সহজ]	১	২	৩	৪	৫
14. Learning communication skills has helped or will help me respect my colleagues [রোগীর সঙ্গে যোগাযোগ দক্ষতা শেখার ফলে আমাকে আমার সহকর্মীদের প্রতি শ্রদ্ধা প্রদর্শনে সহায়তা করেছে বা করবে।]	১	২	৩	৪	৫
15. I find it difficult to trust information about communication skills given to me by nonclinical lecturers [রোগীর সঙ্গে যোগাযোগ দক্ষতা শেখার ক্ষেত্রে চিকিৎসক নন এমন শিক্ষকদের প্রদত্ত তথ্য ও উপকরনে বিশ্বাস করা আমার পক্ষে কঠিন]	১	২	৩	৪	৫
16. Learning communication skills has helped or will help me recognize patients' rights regarding confidentiality and informed consent [চিকিৎসা প্রদান প্রক্রিয়ায় রোগীদের ব্যক্তিগত গোপনীয়তা গুরুত্ব, এবং সেই প্রক্রিয়ায় জেনেশুনে সম্মতির দেওয়ার বিষয়টি রোগীদের এক ধরনের অধিকার। রোগীর সঙ্গে যোগাযোগ দক্ষতা শেখার ফলে এ বিষয়টি উপলব্ধি করতে আমাকে সাহায্য করেছে বা সাহায্য করবে।]	১	২	৩	৪	৫
17. Communication skills teaching would have a better image if it sounded more like a science subject [রোগীর সঙ্গে যোগাযোগ দক্ষতা শিক্ষণ প্রক্রিয়াটি খানিকটা বিজ্ঞান বিষয় পড়ার মত হলে এটি শিক্ষার্থীদের কাছে আরও বেশি গ্রহণযোগ্য হবে।]	১	২	৩	৪	৫
18. When applying for medicine, I thought it was a really good idea to learn communication skills [রোগীর সঙ্গে যোগাযোগ দক্ষতা শেখার বিষয়টি বেশি কাজের। আর এ বিষয়টি মেডিকেল কলেজে ভর্তির আবেদনের সময়ই ভেবেছিলাম।	১	২	৩	৪	৫
19. I don't need good communication skills to be a doctor [একজন চিকিৎসক হওয়ার জন্য আমার রোগীর সঙ্গে যোগাযোগ দক্ষতা শেখার কোনো দরকার নাই।]	১	২	৩	৪	৫
20. I find it hard to admit to having some problems with my communication skills [আমি জানি, আমার যোগাযোগ দক্ষতা খুবই দূর্বল। কিন্তু এই দূর্বলতা বা ঘাটতির কথা স্বীকার করা আমার পক্ষে কঠিন।]	১	২	৩	৪	৫
21. I think it's really useful learning communication skills on the medical degree [আমি মনে করি চিকিৎসা বিজ্ঞানে ডিগ্রির অর্জনের অংশ হিসেবে রোগীর সঙ্গে যোগাযোগ দক্ষতা দক্ষতা শেখার বিষয়টি আসলেই দরকারি।]	১	২	৩	৪	৫
22. My ability to pass exams will get me through medical school rather than my ability to communicate [রোগীর সঙ্গে যোগাযোগ দক্ষতার জন্য নায় বরং চিকিৎসা বিজ্ঞানের বিভিন্ন বিষয়ে জানাশোনার কারনেই আমি মেডিকেল কলেজ থেকে পাশ করতে পারব।]	১	২	৩	৪	৫
23. Learning communication skills is applicable to learning medicine [রোগীর সঙ্গে যোগাযোগ দক্ষতার শেখা চিকিৎসা বিজ্ঞানের অন্যান্য বিষয় শেখার ক্ষেত্রেও খুবই প্রযোজ্য।]	১	২	৩	৪	৫
24. I find it difficult to take communication skills learning seriously [রোগীর সঙ্গে যোগাযোগ দক্ষতার শেখার বিষয়টি আমি মোটেই গুরুত্বের সাথে নেওয়ার বিষয়টি আমার কাছে খুবই কঠিন মনে হয়।]	১	২	৩	৪	৫
25. Learning communication skills is important because my ability to communicate is a lifelong skill [রোগীর সঙ্গে যোগাযোগ দক্ষতার শেখার বিষয়টি খুবই গুরুত্বপূর্ণ। কারন এটি আমার সারা জীবন কাজে লাগবে।]	১	২	৩	৪	৫
26. Communication skills learning should be left to psychology students, not medical students [রোগীর সঙ্গে যোগাযোগ দক্ষতা শেখার বিষয়টি কেবল মনোবিজ্ঞানের শিক্ষার্থীদের ক্ষেত্রে প্রযোজন্য, মেডিকেল শিক্ষার্থীদের ক্ষেত্রে নয়।]	১	২	৩	৪	৫

## ANNEX III

Questionnaire

**Validation of the Bangla version of the Communication Skills Attitude Scale with the medical students of Bangladesh [বাংলাদেশের মেডিক্যাল শিক্ষার্থীদের মাঝে যোগাযোগ দক্ষতা মনোভাব স্কেলের বাংলা সংস্করণের সঠিকতা যাচাই]**

1. **Sociodemographic information (আর্থ‐সামাজিক তথ্য)**
  1.1 Your Age [আপনার বয়স]: _________________________________বছর_
  1.2 Your gender identity [আপনার লৈঙ্গিক পরিচয়]:

1. Male [পুরুষ]
2. Female [নারী]
  1.3 Original residence [আপনার আসল আবাসস্থল]:

1) Rural [গ্রামীন]
2) Upazila level [উপজেলা পর্যায়]
3) District level [জেলা পর্যায়]
4) Division level [বিভাগ পর্যায়]
5) Capital city, Dhaka [রাজধানী ঢাকা]
  1.4 Current residence [আপনার বর্তমান আবাসস্থল]:

1) Hostel of medical college [মেডিক্যাল কলেজের হোস্টেল]
2) Private mess [মেডিক্যাল কলেজের বাইরে কোনো ভাড়া বাড়িতে]
3) Familial residence [পরিবারের কোনো সদস্যের সঙ্গে নিজ বা ভাড়া বাড়িতে]
  1.5 Academic Phases [শিক্ষা স্তর]

1) Phase I [পর্যায়‐১]
2) Phase II [পর্যায়‐২]
3) Phase III [পর্যায়‐৩]
4) Phase IV [পর্যায়‐৪]
  1.6 Academic Year [আপনার শিক্ষাবর্ষ]

1. First Year [প্রথম বর্ষ]
2. Second Year [দ্বিতীয় বর্ষ]
3. Third Year [তৃতীয় বর্ষ]
4. Fourth Year [চতুর্থ বর্ষ]
5. Fifth year [পঞ্চম বর্ষ]
6. Intern [ইন্টার্ন]
  1.7 Type of medical colleges [আপনার মেডিক্যাল কলেজের ধরন]

1. Government [সরকারি]
2. Nongovernment [বেসরকারি]
  1.8 1Monthly income of parents (mother/father) [বাবা মায়ের মাসিক আয়]:
    _________________________________
  1.9 Specialization choice [মেডিকেল কলেজ থেকে পাশ করার পর আপনি যে বিষয়ে বিশেষজ্ঞ হতে চান]

1. Surgery and allied [সার্জারি ও সংশ্লিষ্ট]
2. Medicine and allied [মেডিসিন ও সংশ্লিষ্ট]
3. Gynae and obs [গাইনী ও সংশ্লিষ্ট]
4. Psychiatry [মনোরোগ বিশেষজ্ঞ]
5. Community medicine/public health [কমিউনিটি মেডিসিন/জনস্বাস্থ্য]]
  1.10 Reasons to study medicine [আপনার চিকিৎসা বিদ্যা বিষয়ে পড়ার কারন কী?]

1. Personal passion for caring people [জনগনের সেবা কার জন্য প্রবল আগ্রহ]
2. Family members' demands [পরিবারের সদস্যদের চাপের কারনে]
3. It gives social status [উচ্চ সামাজিক মর্যাদা]
4. It ensures high income [উচ্চ উপার্যনের নিশ্চয়তা]
5. It ensures job/livelihood security [চাকরী বা জীবিকার নিশ্চয়তা]

2. **Communication skills [যোগাযোগ দক্ষতা]**

2.1 Self‐ratings of own communication skills [নিজের যোগাযোগ দক্ষতার স্তর]

1. Poor [খুবই খারাপ]
2. Fair [চলনসই]
3. Average [মোটামুটি]
4. Good [ভালো]
5. Excellent [দুর্দান্ত]

2.2 Do you think that your communication skills need improving? [আপনি কি মনে করেন যে, আপনার যোগাযোগ দক্ষতা বাড়ানো দরকার]

1. Yes [হ্যা]
2. No [না]
3. Not sure [নিশ্চিত নই]

2.3 Barriers to communication skills learning [যোগাযোগ শেখার ক্ষেত্রে বাধাসমূহ]

1. Not having any academic subject to learning communication skills at medical college [যোগাযোগ দক্ষতা শেখার জন্য মিডিক্যাল কলেজে কোনো কোর্স নেই।]
2. Not aware of importance of learning communication skills [একজন চিকিৎসকের যোগাযোগ দক্ষতা শেখার প্রয়োজনীয়তা সম্পর্কে জানা নেই।]
3. Think that there is nothing to learning to communication [আমি মনে করি যে, যোগাযোগ দক্ষতার কিছু নেই।]
4. Not emphasis given in medical training to learning communication skills [মেডিকেল কলেজে পঠন‐পাঠন প্রক্রিয়া যোগাযোগ দক্ষতা শেখার ওপর তেমন গুরুত্ব দেওয়া হয় না]
5. Others (specify) [অন্যান্য, নির্দিষ্ট করুন]

**Communication Skills Attitude Scale (CSAS) [যোগাযোগ দক্ষতার প্রতি মনোভাব স্কেল]** ^34^

Please read the following statements about communication skills learning. Indicate whether you disagree or agree with all of the statements according to the following scale: 1 = *strongly disagree*, 2 = disagree, 3 = *neutral*, 4 = *agree*, and 5 = *strongly agree*. [রোগীর সঙ্গে যোগাযোগ [‘কথা বলা, শোনা, অঙ্গভঙ্গি, মুখোভঙ্গি ও কণ্ঠস্বর ইত্যাদি] দক্ষতা শিক্ষণ বিষয়ে নিম্নলিখিত বিবৃতিগুলো দয়া করে পড়ুন। নিচের স্কেলে উল্লেখিত যেসব বিবৃতির সঙ্গে আপনি দ্বিমত বা একমত পোষণ করেন তা নির্দেশ করুন। আর সেক্ষেত্রে টিক চিহ্ন ব্যবহার করুন। এখানে, ১= দৃঢ়ভাবে দ্বিমত পোষণ করি, ২ = দ্বিমত পোষণ করি, ৩ = নিরপেক্ষ, ৪ = একমত পোষণ করি, এবং ৫ = দৃঢ়ভাবে একমত পোষণ করি]

**Table:**

	Strongly disagree [দৃঢ়ভাবে দ্বিমত পোষণ করি]	Disagree [দ্বিমত পোষণ করি]	Neutral [নিরপেক্ষ/ নিশ্চিত নিই/বুঝতে পারছি না]	Agree [একমত পোষণ করি]	Strongly Agree [দৃঢ়ভাবে একমত পোষণ করি]
	১	২	৩	৪	৫
1. To be a good doctor I must have good communication skills [একজন ভালো ডাক্তার হতে হলে আমার ভালো যোগাযোগ দক্ষতা দক্ষতা থাকতে হবে।]	১	২	৩	৪	৫
2. I can't see the point in learning communication skills [একজন ডাক্তারের তার রোগীর সঙ্গে ভালো যোগাযোগ দক্ষতা শেখার দরকার আছে বলে আমি মনে করি না।	১	২	৩	৪	৫
3. Nobody is going to fail their medical degree for having poor communication skills [রোগীর সঙ্গে ভালো যোগাযোগ দক্ষতা না থাকার কারনে মেডিকেল কলেজের পরিক্ষায় কোনো শিক্ষার্থী অকৃতকার্য হয় না।]	১	২	৩	৪	৫
4. Developing my communication skills is just as important as developing my knowledge of medicine. [রোগীর সঙ্গে যোগাযোগ দক্ষতা বাড়ানো চিকিৎসাবিদ্যায় প্রয়োজনীয় জ্ঞান অর্জনের মতই সমানভাবে গুরুত্বপূর্ণ।]	১	২	৩	৪	৫
5. Learning communication skills has helped or will help me respect patients [রোগীর সঙ্গে ভালো যোগাযোগ দক্ষতা শেখা আমাকে রোগীর প্রতি সম্মান প্রদর্শনে সহায়তা করেছে বা ভবিষ্যতে করবে।]	১	২	৩	৪	৫
6. I haven't got time to learn communication skills [আমি রোগীর সঙ্গে ভালোভাবে যোগাযোগ দক্ষতা শেখার সময় পায়নি।]	১	২	৩	৪	৫
7. Learning communication skills is interesting [রোগীর সঙ্গে ভালোভাবে যোগাযোগ দক্ষতা শেখার বিষয়টি বেশ আকর্ষণীয়]	১	২	৩	৪	৫
8. I can't be bothered to turn up to sessions on communication skills [রোগীর সঙ্গে ভালো যোগাযোগ দক্ষতা শেখার কোনো ক্লাশ বা প্রশিক্ষণ কর্মশালয় অংশগ্রহণে আমার কোনো আগ্রহ নেই।]	১	২	৩	৪	৫
9. Learning communication skills has helped or will help facilitate my team‐working skills [রোগীর সঙ্গে ভালো যোগাযোগ দক্ষতা শেখা আমাকে অন্যের সঙ্গে একযোগে কাজ করতে সহায়তা করেছে বা করবে।]	১	২	৩	৪	৫
10. Learning communication skills has improved my ability to communicate with patients [যোগাযোগ দক্ষতা শেখার ফলে আমার রোগীর সঙ্গে যোগাযোগ সক্ষমতা বেড়েছে।	১	২	৩	৪	৫
11. Communication skills teaching states the obvious and then complicates it [যোগাযোগ দক্ষতা শিক্ষণ প্রক্রিয়ায় মূলত জানা বিষয়গুলোই তুলে ধরা হয়। কিন্তু এসব বিষয় শেখার ফলে, রোগীর সঙ্গে বলা ও শোনার স্বাভাবিক বিষয়টি বেশ জটিলও হয়ে যায়।]	১	২	৩	৪	৫
12. Learning communication skills is fun [যোগাযোগ দক্ষতা শেখার বিষয়টি বেশ আনন্দদায়ক]	১	২	৩	৪	৫
13. Learning communication skills is too easy রোগীর সঙ্গে যোগাযোগ দক্ষতা শেখার বিষয়টি বেশ সহজ]	১	২	৩	৪	৫
14. Learning communication skills has helped or will help me respect my colleagues [রোগীর সঙ্গে যোগাযোগ দক্ষতা শেখার ফলে আমাকে আমার সহকর্মীদের প্রতি শ্রদ্ধা প্রদর্শনে সহায়তা করেছে বা করবে।]	১	২	৩	৪	৫
15. I find it difficult to trust information about communication skills given to me by nonclinical lecturers [রোগীর সঙ্গে যোগাযোগ দক্ষতা শেখার ক্ষেত্রে চিকিৎসক নন এমন শিক্ষকদের প্রদত্ত তথ্য ও উপকরনে বিশ্বাস করা আমার পক্ষে কঠিন]	১	২	৩	৪	৫
16. Learning communication skills has helped or will help me recognize patients' rights regarding confidentiality and informed consent [চিকিৎসা প্রদান প্রক্রিয়ায় রোগীদের ব্যক্তিগত গোপনীয়তা গুরুত্ব, এবং সেই প্রক্রিয়ায় জেনেশুনে সম্মতির দেওয়ার বিষয়টি রোগীদের এক ধরনের অধিকার। রোগীর সঙ্গে যোগাযোগ দক্ষতা শেখার ফলে এ বিষয়টি উপলব্ধি করতে আমাকে সাহায্য করেছে বা সাহায্য করবে।]	১	২	৩	৪	৫
17. Communication skills teaching would have a better image if it sounded more like a science subject [রোগীর সঙ্গে যোগাযোগ দক্ষতা শিক্ষণ প্রক্রিয়াটি খানিকটা বিজ্ঞান বিষয় পড়ার মত হলে এটি শিক্ষার্থীদের কাছে আরও বেশি গ্রহণযোগ্য হবে।]	১	২	৩	৪	৫
18. When applying for medicine, I thought it was a really good idea to learn communication skills [রোগীর সঙ্গে যোগাযোগ দক্ষতা শেখার বিষয়টি বেশি কাজের। আর এ বিষয়টি মেডিকেল কলেজে ভর্তির আবেদনের সময়ই ভেবেছিলাম।	১	২	৩	৪	৫
19. I don't need good communication skills to be a doctor [একজন চিকিৎসক হওয়ার জন্য আমার রোগীর সঙ্গে যোগাযোগ দক্ষতা শেখার কোনো দরকার নাই।]	১	২	৩	৪	৫
20. I find it hard to admit to having some problems with my communication skills [আমি জানি, আমার যোগাযোগ দক্ষতা খুবই দূর্বল। কিন্তু এই দূর্বলতা বা ঘাটতির কথা স্বীকার করা আমার পক্ষে কঠিন।]	১	২	৩	৪	৫
21. I think it's really useful learning communication skills on the medical degree [আমি মনে করি চিকিৎসা বিজ্ঞানে ডিগ্রির অর্জনের অংশ হিসেবে রোগীর সঙ্গে যোগাযোগ দক্ষতা দক্ষতা শেখার বিষয়টি আসলেই দরকারি।]	১	২	৩	৪	৫
22. My ability to pass exams will get me through medical school rather than my ability to communicate [রোগীর সঙ্গে যোগাযোগ দক্ষতার জন্য নায় বরং চিকিৎসা বিজ্ঞানের বিভিন্ন বিষয়ে জানাশোনার কারনেই আমি মেডিকেল কলেজ থেকে পাশ করতে পারব।]	১	২	৩	৪	৫
23. Learning communication skills is applicable to learning medicine [রোগীর সঙ্গে যোগাযোগ দক্ষতার শেখা চিকিৎসা বিজ্ঞানের অন্যান্য বিষয় শেখার ক্ষেত্রেও খুবই প্রযোজ্য।]	১	২	৩	৪	৫
24. I find it difficult to take communication skills learning seriously [রোগীর সঙ্গে যোগাযোগ দক্ষতার শেখার বিষয়টি আমি মোটেই গুরুত্বের সাথে নেওয়ার বিষয়টি আমার কাছে খুবই কঠিন মনে হয়।]	১	২	৩	৪	৫
25. Learning communication skills is important because my ability to communicate is a lifelong skill [রোগীর সঙ্গে যোগাযোগ দক্ষতার শেখার বিষয়টি খুবই গুরুত্বপূর্ণ। কারন এটি আমার সারা জীবন কাজে লাগবে।]	১	২	৩	৪	৫
26. Communication skills learning should be left to psychology students, not medical students [রোগীর সঙ্গে যোগাযোগ দক্ষতা শেখার বিষয়টি কেবল মনোবিজ্ঞানের শিক্ষার্থীদের ক্ষেত্রে প্রযোজন্য, মেডিকেল শিক্ষার্থীদের ক্ষেত্রে নয়।]	১	২	৩	৪	৫
